# James Lind Alliance Priority Setting Partnership in co-existing dementia and hearing conditions: a research agenda defined by people with lived experience and healthcare professionals

**DOI:** 10.1093/ageing/afaf191

**Published:** 2025-07-06

**Authors:** Eithne Heffernan, Sian Calvert, Tom Dening, Emma E Broome, Ruth Spriggs, Nahid Ahmad, Natalie Lerigo-Smith, Helen Henshaw

**Affiliations:** National Institute for Health and Care Research (NIHR), Nottingham Biomedical Research Centre, Nottingham, UK; Hearing Sciences, Mental Health and Clinical Neurosciences, Faculty of Medicine and Health Sciences, School of Medicine, University of Nottingham, Nottingham, UK; National Institute for Health and Care Research (NIHR), Nottingham Biomedical Research Centre, Nottingham, UK; Hearing Sciences, Mental Health and Clinical Neurosciences, Faculty of Medicine and Health Sciences, School of Medicine, University of Nottingham, Nottingham, UK; Mental Health and Clinical Neurosciences, Faculty of Medicine and Health Sciences, School of Medicine, University of Nottingham, Nottingham, UK; National Institute for Health and Care Research (NIHR), Nottingham Biomedical Research Centre, Nottingham, UK; Hearing Sciences, Mental Health and Clinical Neurosciences, Faculty of Medicine and Health Sciences, School of Medicine, University of Nottingham, Nottingham, UK; National Institute for Health and Care Research (NIHR), Nottingham Biomedical Research Centre, Nottingham, UK; Hearing Sciences, Mental Health and Clinical Neurosciences, Faculty of Medicine and Health Sciences, School of Medicine, University of Nottingham, Nottingham, UK; James Lind Alliance, NIHR, Southampton, UK; National Institute for Health and Care Research (NIHR), Nottingham Biomedical Research Centre, Nottingham, UK; Hearing Sciences, Mental Health and Clinical Neurosciences, Faculty of Medicine and Health Sciences, School of Medicine, University of Nottingham, Nottingham, UK; National Institute for Health and Care Research (NIHR), Nottingham Biomedical Research Centre, Nottingham, UK; Hearing Sciences, Mental Health and Clinical Neurosciences, Faculty of Medicine and Health Sciences, School of Medicine, University of Nottingham, Nottingham, UK

**Keywords:** dementia, mild cognitive impairment, hearing loss, tinnitus, vestibular disorders, older people

## Abstract

**Background:**

Dementia and hearing conditions are both major public health concerns. Most people living with dementia also live with hearing conditions (e.g. hearing loss, tinnitus, vestibular disorders). Furthermore, hearing loss may be a risk factor for dementia. There is a critical need for research to explain the association between dementia and hearing conditions and to optimise assessments and treatments for this co-morbidity.

**Objective:**

This James Lind Alliance (JLA) Priority Setting Partnership (PSP) aimed to develop an agenda for research about dementia and hearing conditions by systematically identifying unanswered research questions that are prioritised by people living with these conditions and professionals from healthcare and social care.

**Methods:**

Participants were people living with hearing conditions and/or dementia, supporters (e.g. carers, relatives), clinicians, and social care professionals. A survey (*N* = 404) gathered 47 research questions proposed by participants. An evidence-checking process confirmed that these questions were unanswered. A second survey (*N* = 560) produced a shortlist of 16 questions. At a final workshop (*N* = 19), the top 10 questions were prioritised.

**Results:**

The prioritised research questions spanned diverse topics, including training for clinicians about this co-morbidity, routine health checks that incorporate hearing and cognition, dementia risk reduction strategies for people living with hearing loss, and potential mechanisms underlying the link between hearing loss and dementia.

**Conclusion:**

This novel JLA PSP was the first to identify research priorities for two different, yet co-morbid, health conditions. It has important implications for researchers, funders, commissioners, and clinicians, particularly those working with older adults who have multiple long-term conditions.

## Key Points

- First James Lind Alliance Priority Setting Partnership (PSP) to bring together two different health areas: dementia and hearing conditions.
- The PSP was overseen by a steering group including people living with dementia and/or hearing conditions and clinicians.
- The process was adapted to support the involvement of people with dementia and/or hearing conditions and sign language users.
- It confirmed the research priorities of experts by experience and clinicians to help inform the national research direction.
- Priority research areas included risk reduction, screening, diagnosis, clinician training, interventions and causal mechanisms.

## Background

Over 430 million people worldwide are estimated to live with hearing loss, whilst 55 million are estimated to live with dementia [1, 2]. In the United Kingdom, approximately 18 million people live with hearing loss and almost one million people live with dementia [3, 4]. It is also estimated that around 20% of those aged over 50 years globally live with Mild Cognitive Impairment (MCI) [5]. MCI is characterised by cognitive decline beyond normal ageing and can be a precursor for the development of dementia [6]. The prevalence of these progressive conditions is predicted to grow as life expectancy increases [1, 2]. Most people living with dementia or MCI also live with hearing loss and/or additional hearing conditions (e.g. vestibular disorders, tinnitus and hyperacusis). Both dementia and hearing conditions are major public health concerns. Dementia is a leading cause of death in the United Kingdom and has an estimated economic impact of £42.5 billion a year, which includes unpaid care, social care, healthcare and quality of life costs [4]. Untreated hearing loss costs the UK economy £25.5 billion annually due to its detrimental impact on quality of life, healthcare and productivity [7, 8].

Individually, dementia and hearing conditions can have numerous negative consequences, including impaired communication and concentration, social isolation, depressive symptoms, stigma and third-party disability [9, 10]. These consequences are exacerbated when people experience dementia and hearing conditions co-morbidly, which can be further impacted by the prolonged time it can take to obtain diagnoses and interventions [11, 12]. While neither condition can yet be cured, management options are available for dementia (e.g. cognitive rehabilitation, reminiscence therapy) and hearing conditions (e.g. hearing aids, cochlear implants). However, further research is needed to identify the optimal management approaches for people who live with both dementia and hearing conditions [9]. Research is also needed to establish the optimal approaches to assessing hearing and cognition in this population. In recent years, cognitive tests suitable for people living with hearing loss have been developed [13]. Modifications to hearing assessments for people living with dementia have been proposed, but further investigations are required to determine their effectiveness [14, 15].

The 2024 Lancet Commission on Dementia included untreated hearing loss among the potentially modifiable risk factors for the development of dementia [16]. However, the evidence linking hearing loss to an increased risk of dementia remains inconclusive [17–19]. It is possible that managing hearing loss could help to reduce, prevent or delay cognitive decline, especially in high-risk groups [16, 20]. In people already living with MCI or dementia, managing hearing loss has the potential to improve behavioural and psychological symptoms (e.g. impaired communication, social isolation), thereby enhancing overall quality of life [21, 22]. However, people living with dementia may require additional support and adaptations to use hearing interventions [23].

People living with dementia and hearing conditions have significant unmet needs and there remain many unanswered questions about this co-morbidity, which should be addressed by research. Therefore, the James Lind Alliance (JLA) Priority Setting Partnership (PSP) in co-existing dementia and hearing conditions was formed. JLA PSPs are an established means of bringing together people with lived experience, supporters (e.g. relatives, spouses), and clinicians on an equal footing to decide the research agenda for a specific health condition or area. Research agendas are often determined by the research community and industry and thus may not reflect the needs of those who live with health conditions, or who provide care for these conditions. PSPs can bridge the gap between different stakeholders by fostering collaboration and ensuring that research is driven by the priorities of the ‘end users’, particularly people with lived experience and clinicians. In doing so, PSPs help to reduce research waste and increase the likelihood that study findings will be implemented in practice. Previous PSPs have identified research priorities in dementia, mild-to-moderate hearing loss, tinnitus and hyperacusis [24–28]. The current JLA PSP in co-existing dementia and hearing conditions is the first to bring together two different health conditions. It aimed to develop an agenda for research about co-existing dementia and hearing conditions by identifying unanswered research questions that are important to people with lived experience and professionals from healthcare and social care.

## Methods

The PSP project (March 2023–September 2024) was conducted in accordance with published JLA methods [29]. The protocol was published on the PSP webpage [30].

### Management and scope

The PSP Lead, PSP Coordinator, and JLA Advisor worked in partnership with a UK-based steering group (*N* = 17), which included people living with dementia and/or hearing conditions; representatives from charities for hearing conditions and dementia, and clinicians (Supplementary Appendix 1). They met approximately every 7 weeks to review project progress, approve project documents, and agree on the next steps. The meetings were chaired independently by the JLA Advisor, who helped ensure that the process was fair and transparent and that people with lived experience and clinicians had equal input. A partner group, which included clinicians, researchers, Patient and Public Involvement leads, and government representatives, supported project promotion and dissemination.

The scope of this PSP was to identify and prioritise unanswered research questions about dementia, MCI, and hearing conditions, particularly hearing loss, tinnitus, hyperacusis and vestibular disorders. This could include questions about risk reduction, diagnosis and treatment, provided that they were relevant to both hearing conditions and dementia or MCI.

### Ethical approval

Ethical approval was not required, as the PSP constituted a public consultation and engagement activity [25, 31].

### Survey 1: generating the questions

An initial survey (November 2023–January 2024) collected potential research questions about co-existing dementia and hearing conditions. The respondents were people with lived experience of dementia/MCI and/or hearing conditions, supporters, clinicians and social care professionals. Researchers who did not belong to one of these categories were not eligible to participate. The survey was developed with the steering group to ensure that it was accessible to diverse respondents, including laypeople and professionals. Respondents proposed questions for future research about dementia and hearing conditions, including questions about risk reduction, diagnosis, treatment and any other topics that they deemed important. They also provided demographic information.

The survey was available in various formats (e.g. digital, postal). It was translated into British Sign Language (BSL) for the Deaf community. All respondents could request an alternative means of providing their responses (e.g. telephone, teleconferencing) by contacting the PSP coordinator. Respondents were recruited via a range of channels, primarily in the United Kingdom, including an internal participant database, steering and partner group networks, social media, charities (e.g. RNID), professional societies (e.g. British Society of Audiology), groups for people with lived experience (e.g. Dementia Engagement and Empowerment Project), newsletters and blogs (e.g. INTERDEM), magazine articles (e.g. British Academy of Audiology Magazine), and conferences (e.g. Alzheimer Europe Conference).

The research questions proposed by respondents were analysed to develop a list of summary questions. Summary questions should be clear and succinct questions that capture the topics and themes proposed by people with lived experience, supporters and professionals [29]. Summary questions typically require additional refinement following the PSP process to become specific research questions [29, 32]. The analysis was conducted by the research team and reviewed by steering group members and the JLA advisor. First, duplicate and out-of-scope questions were removed. The remaining questions were coded into categories (e.g. causal mechanisms, treatment, diagnosis). Subsequently, similar or related questions were collated to form the summary questions. The summary questions were written in plain English in collaboration with the steering group to ensure accessibility to lay audiences.

### Evidence checking: verifying the questions

The research team examined the existing evidence for each summary question to determine whether any questions had been answered, or partially answered, by previous research. The existing evidence comprised systematic reviews and clinical guidelines that were relevant and recent (i.e. no more than 3 years old). To identify relevant systematic reviews, the Cochrane and Medline databases were searched using key terms (e.g. ‘dementia’, ‘hearing loss’, ‘hyperacusis’, ‘tinnitus’ and ‘vestibular’). Clinical guidelines were identified via the National Institute for Health and Care Excellence, the Royal Colleges, and steering group recommendations. Questions were considered answered if there was either a review or guideline that provided a definitive conclusion and did not indicate that additional research was needed to answer the question. A subgroup of steering group members assisted with this process and approved its outcomes.

### Survey 2: identifying priority questions

In a second survey (June–August 2024), respondents reviewed the list of summary questions and selected the 10 questions they considered most important to them. The purpose was to develop a shortlist of priority summary questions to be taken forward to the final workshop. The survey was available in the same formats (e.g. digital, postal, BSL) and was disseminated using the same channels as the first survey. In addition, it was shared with respondents from the first survey who had expressed an interest in the second survey.

### Final workshop: agreeing on the top 10 questions

The final stage was a workshop (September 2024) to review the shortlist of priority summary questions and to decide by consensus the top 10 questions about co-existing dementia and hearing conditions. The steering group, JLA advisor and research team co-designed the workshop format. This included holding the workshop online to be inclusive of people in different geographical regions and working clinicians. To be inclusive of people living with hearing loss and/or dementia, the workshop length was reduced by having fewer questions to review compared to previous PSPs.

The workshop attendees were survey respondents who expressed an interest in the workshop. Maximum variation sampling was used to recruit attendees who represented a range of groups (i.e. people with lived experience, supporters, healthcare professionals, social care professionals) and who had diverse demographic characteristics (e.g. gender, age group, ethnicity, BSL use). The workshop was led by four JLA facilitators. They were supported by the research team, a technical advisor, and two BSL interpreters.

Prior to the workshop, attendees were sent information videos about dementia and hearing conditions, JLA PSPs, and the workshop, as well as an information pack including the shortlist of priority summary questions, delegate list, and workshop agenda. During the workshop, attendees were divided into four groups and participated in three discussion sessions. These sessions involved completing ranking and feedback exercises using the Nominal Group Technique: an established structured group process that encourages participation from all individuals to reach a decision by consensus [33]. In the first session, attendees discussed their two highest-ranked and two lowest-ranked questions. In the second and third sessions, they discussed how the 16 questions should be ranked. The second session results were reviewed before ranking the questions in the third session. Following the third session, the facilitators collated the rankings from the different groups to identify the top 10 questions, which were then presented to the attendees.

## Results

**Figure 1:**
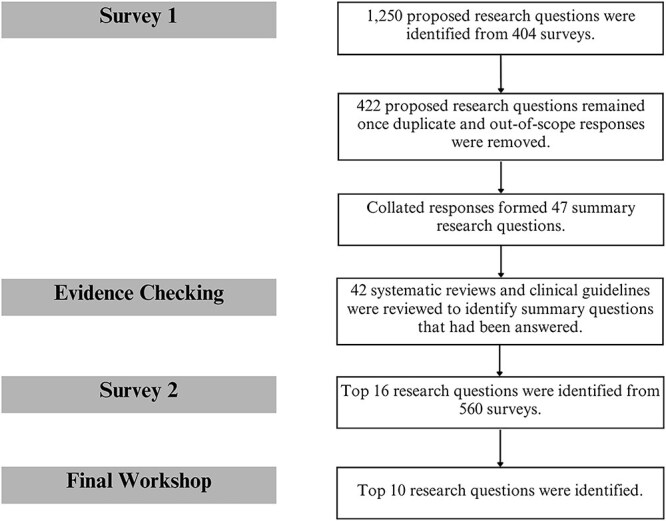
Flow diagram of the PSP process.

Figure 1 shows each stage of the PSP process, from generating the initial questions to agreeing on the top 10 questions.

### Survey 1: generating the questions

The PSP received 578 surveys. Surveys that did not contain nominated research questions or that were completed by researchers were removed. The remaining 404 completed surveys were analysed. The analysis is available on the PSP webpage [34]. Respondents (Table 1) could belong to multiple groups. For example, a respondent could be a clinician and living with a hearing condition. The survey gathered 1250 suggested research questions. Once out-of-scope and duplicate responses were removed, the 422 remaining questions were categorised. These questions were then collated to form 47 summary questions (Supplementary Appendix 2), which represented the following categories: awareness (*n* = 2), diagnosis (*n* = 13), health services (*n* = 1), interaction with other conditions (*n* = 2), causal mechanisms (*n* = 4), prognosis (*n* = 5), risk (*n* = 4), social care (*n* = 3), and treatment (*n* = 13).

**Table 1:** Demographic information of the survey respondents.

Respondents^a^	Survey 1	Survey 2
All respondents	404	560
People living with a hearing condition	296 (73.27%)	455 (81.25%)
Hearing loss	241	424
Tinnitus	156	239
Vestibular disorders	45	77
Hyperacusis	41	41
People living with MCI or dementia	32 (7.92%)	51 (9.11%)
Alzheimer’s disease	8	9
Vascular dementia	4	1
Mixed dementia	4	7
Frontotemporal dementia	1	1
Lewy body dementia	1	1
MCI	3	8
MCI or dementia without a formal diagnosis	11	24
Supporters (e.g. family carers, partners, relatives)	264 (65.35%)	377 (67.32%)
Hearing condition supporters	221	324
MCI or dementia supporters	186	257
Hearing conditions and MCI or dementia supporters	143	204
Health and social care professionals	169 (41.83%)	175 (31.25%)
Audiologists	82	33
Nurses	19	48
Social care professionals	19	25
Medical doctors	18	14
Psychologists and therapists	9	7
Occupational therapists	3	5
Other (e.g. paramedic, pharmacist, social prescriber)	19	43
Gender	–	–
Man	104	153
Woman	293	402
Non-binary person	3	1
Prefer not to say	3	3
Age group	–	–
18–29	34	16
30–39	34	27
40–49	40	50
50–59	72	83
60–69	93	150
70–79	96	167
80 and above	33	65
Under-served groups	–	–
Other disability or health condition	112	204
Member of an ethnic minority group	66	25
Lives in a rural or remote area	49	75
Other mental health condition	43	62
Lives on a low income	32	62
Member of the LGBTQ+ community	17	20
Member of the Deaf community	9	15
Unrecognised or no educational qualifications	4	11
Was or is unhoused or houseless	4	3
Member of another underserved group	14	7

^a^Respondents could self-identify as belonging to one group or to multiple groups (e.g. supporter of someone living with MCI or dementia, supporter of someone living with a hearing condition, health and social care professional, person living with hearing conditions, person living with MCI or dementia) via the demographic section of the survey.

### Evidence checking: verifying the questions

The literature search retrieved 1089 articles. Thirty-two relevant and recent systematic reviews and 10 clinical practice guidelines were identified and examined (Supplementary Appendix 3). This review confirmed that none of the 47 summary questions had been answered or partially answered by previous research. Therefore, all 47 were included in the second survey.

### Survey 2: identifying priority questions

The PSP received 563 surveys. Surveys that did not contain a top 10 list of questions or that were completed by researchers were excluded. Therefore, 560 surveys were included in the analysis. The analysis identified a shortlist of priority summary questions to be discussed at the workshop (Supplementary Appendix 2). This shortlist consisted of the 15 highest-ranked questions from the survey. There were considerably fewer respondents living with dementia (*n* = 51) compared to the other respondent groups. Therefore, their rankings were reviewed to ensure that they were not overshadowed by the other groups. This led to the inclusion of a 16th summary question in the shortlist (Question 10 in Supplementary Appendix 2), which had been ranked highly by people living with dementia. The 16 questions represented the following categories: diagnosis (*n* = 5), causal mechanisms (*n* = 4), risk (*n* = 3), treatment (*n* = 3), and prognosis (*n* = 1).

### Final workshop: agreeing on the top 10 questions

There were 19 attendees (Table 2) based in the United Kingdom, including people living with hearing conditions (*n* = 14) and/or MCI or dementia (*n* = 6), supporters of someone living with a hearing condition (*n* = 8) and/or MCI or dementia (*n* = 3) and health and social care professionals (*n* = 9), such as general practitioners, audiologists and nurses. One person used BSL as their primary language. Table 3 shows the top 10 questions decided by consensus during the workshop. In the first session, several attendees reported that all 16 questions were important, and it was difficult to select their lowest-ranked questions. Following the second session, the four groups had similar top 10 selections. Following the third session, there was even greater similarity between the top 10 selections of the groups.

**Table 2:** Demographic information of the workshop attendees.

Index	Gender	Group(s)^a^
1	Female	Person living with hearing loss
2	Female	Supporter of someone living with MCI or dementia and a hearing condition
3	Male	Person living with dementia and hearing loss
4	Female	Person living with hearing loss; Supporter of someone living with a hearing condition
5	Male	Supporter of someone living with MCI or dementia; Social care professional
6	Female	Supporter of someone living with a hearing condition; Healthcare professional
7	Female	Person living with hearing loss and a vestibular disorder; Healthcare professional
8	Female	Person living with hearing loss and a vestibular disorder; Health care professional; Social care professional
9	Female	Person living with tinnitus; Healthcare professional
10	Male	Person living with hearing loss
11	Female	Supporter of someone living with a hearing condition; Healthcare professional
12	Male	Person living with hearing loss and tinnitus; Supporter of someone living with a hearing condition; Healthcare professional
13	Male	Person living with hearing loss, tinnitus and dementia
14	Female	Supporter of someone living with a hearing condition; Healthcare professional
15	Male	Person living with hearing loss, tinnitus, a vestibular disorder and MCI; Supporter of someone living with MCI or dementia and a hearing condition
16	Male	Person living with hearing loss and MCI; Healthcare professional
17	Female	Person living with hearing loss, tinnitus, hyperacusis and MCI; Supporter
18	Male	Person living with hearing loss, tinnitus and MCI
19	Male	Member of the Deaf community; Charity Representative

^a^Attendees could self-identify as belonging to one group or to multiple groups (e.g. supporter of someone living with MCI or dementia, supporter of someone living with a hearing condition, health and social care professional, person living with hearing conditions, person living with MCI or dementia) via the demographic section of the survey.

**Table 3:** The JLA PSP’s top 10 priorities for research about co-existing dementia and hearing conditions.^a^

Rank	Question
1	What actions can people who have hearing loss take to reduce their risk of developing dementia?
2	Can the early detection and management of cognitive or hearing difficulties for people lead to better outcomes or treatments?
3	Does hearing loss increase dementia risk, and if so, what are the underlying mechanisms or causes (e.g. vascular disease, biological or neurological mechanisms)?
4	What training would help health professionals provide appropriate support and communicate effectively with people living with both dementia and hearing conditions?
5	Is the link between hearing loss and dementia risk impacted by other factors (e.g. personality, lifestyle, additional health conditions, or social isolation)?
6	Does dementia impact hearing, or does hearing impact dementia (e.g. severity, rate of progression)?
7	Can auditory and cognitive training be used by people with hearing loss to improve cognition and/or reduce the risk of developing dementia?
8	Should routine health checks in adults assess both hearing and cognition?
9	Are there hereditary/genetic factors that increase the likelihood of developing co-existing dementia and hearing conditions?
10	What is the best way for primary care professionals (e.g. general practitioners) to support the assessment of dementia and hearing conditions and to improve their understanding of the link between them?

^a^Hearing conditions and hearing difficulties include hearing loss, tinnitus, hyperacusis, and vestibular disorders.

## Discussion

There are many unanswered questions surrounding the co-occurrence of dementia and hearing conditions, with a notable lack of evidence-based clinical guidelines for the diagnosis and management of this co-morbidity [9]. The present JLA PSP engaged people with lived experience, supporters and professionals from health and social care to identify priority questions for future research about co-existing dementia and hearing conditions. The resulting research agenda will help ensure that future dementia and hearing research not only advances scientific knowledge but also translates into meaningful outcomes for those directly impacted by these conditions. The agenda reveals critical gaps in current knowledge where clinical advancements could significantly improve outcomes for patients and families, as well as healthcare resource utilisation. The agenda can inform the national research direction, such as through the NIHR rolling call for studies addressing JLA PSP priorities [35].

The agenda encompasses diverse topics such as awareness, causal mechanisms, diagnosis and treatment. Several priority research questions concern risk and risk reduction, including the top-ranked question: *‘What actions can people who have hearing loss take to reduce their risk of developing dementia?’* Other priority questions pertain to people who already live with both dementia and a hearing condition: a population that is often overlooked when the association between dementia and hearing is discussed. Furthermore, whilst some priority questions focus specifically on hearing loss, others have a broader focus on hearing conditions (e.g. hearing loss, tinnitus, hyperacusis) generally. In addition, several priority questions have significant clinical relevance and implications. These include questions about improving clinicians’ understanding and assessment of dementia and hearing conditions, as well as their communication with, and support for, people living with these conditions. They also include questions about incorporating cognition and hearing into routine health checks and about the impact of the early detection and management of cognitive or hearing difficulties on outcomes and care. Addressing these questions could lead to more effective, timely and coordinated care pathways that better meet the needs of people living with these conditions and their supporters.

This is the first PSP to bring two different conditions together and its results differ from previous PSPs that examined dementia or hearing conditions in isolation [26, 28]. For example, several research priorities from the current PSP concerned risk reduction and improving clinicians’ awareness and training, whilst the previous dementia PSP placed greater emphasis on post-diagnostic support and the impact on supporters. Furthermore, the mild-to-moderate hearing loss PSP had a greater number of research priorities concerning hearing aids than the present PSP. It is vital to adopt an approach that accounts for multi-morbidity when determining research priorities and conducting research to optimise care [31, 36].

A strength of this PSP was that it entailed a methodologically rigorous and transparent process. It followed an independently developed method, culminating in a workshop led by independent JLA facilitators. It was overseen by a steering group with representatives from key stakeholder groups, including people with lived experience, supporters, clinicians and charity partners. These stakeholder groups were also well represented at the final workshop. Another strength was the substantial number of survey responses received. However, a relatively small number of people living with MCI or dementia completed the surveys, despite extensive recruitment efforts. Dementia and MCI are considerably less prevalent than hearing conditions. Many studies report difficulties recruiting participants who live with dementia, with just 1% of people who could participate in UK dementia trials doing so [37]. The number of people living with dementia in this PSP is comparable to previous studies that used consensus methods with dementia stakeholders [38, 39]. Furthermore, it is possible that the people living with MCI and dementia who contributed to this PSP are not representative of the broader population living with these conditions. For example, they may have had greater social support or cognitive function than those who were not involved, which could affect the generalisability of the findings.

This novel PSP has produced an agenda for future research about dementia and hearing conditions that focuses on the questions most valued by people with lived experience, supporters and professionals from health and social care. By directing attention to clinically relevant research questions, we aim to reduce research waste and promote meaningful advancements in evidence-based healthcare for older adults. The findings have important implications not only for researchers, funders and commissioners, but also for policymakers and clinicians, by highlighting key areas of concern for the growing number of people affected by dementia and hearing conditions.

## Supplementary Material

Appendix_1_afaf191

Appendix_2_afaf191

Appendix_3_afaf191
