# Supplementary material for: James Lind Alliance Priority Setting Partnership in co-existing dementia and hearing conditions: a research agenda defined by people with lived experience and healthcare professionals
Source: Age Ageing. 2025 Jul 6;54(7):afaf191. doi: 10.1093/ageing/afaf191 (PMC12229087; doi:10.1093/ageing/afaf191)
Supplement: Appendix_1_afaf191 [file appendix_1_afaf191.docx]

## **James Lind Alliance Priority Setting Partnership in Co-existing Dementia and Hearing Conditions: A Research Agenda defined by People with Lived Experience and Healthcare Professionals.**

## **Supplementary Materials**

### **List of Contents**

Appendix 1: Priority Setting Partnership Steering Group Members……………….Pages 2-3

Appendix 2: List of 47 Summary Research Questions……………………………..Pages 4-6

Appendix 3: Evidence Checking Process………………………………………….Pages 7-10

### **Appendix 1**

### **Priority Setting Partnership Steering Group Members**

The steering group included four people living with lived experience of dementia and/or hearing conditions; a supporter of someone living with dementia and hearing loss; representatives from charities for hearing conditions and dementia, a representative from the British Society of Audiology, and seven healthcare professionals (i.e. audiologists, psychiatrists, a general practitioner, and a consultant audiovestibular physician).

**Steering Group Members:**

Anna Smith, Head of Involvement, Alzheimer’s Society

Bhavisha Parmar, Postdoctoral Research Audiologist and Lecturer- University of Cambridge, UCL Ear Institute.

Dr Devina Maru, General Practitioner with specialist interest in ENT

Dr Dolapo Oseji, Specialist Registrar with Northamptonshire Healthcare NHS Foundation Trust.

Dr Hannah Semeraro, Insight and Evidence Team Lead, Royal National Institute for Deaf People (RNID).

Dr Kathryn Fackrell, Senior Research Fellow, The University of Nottingham, Faculty of Medicine and Health Sciences

Dr Rohani Omar, Consultant Audiovestibular Physician and UCL Honorary Associate Professor, University College London Hospitals NHS Foundation Trust

Elaine Beardsley-Turton, Expert by experience

Jack Stancel-Lewis, Audiologist, University of Nottingham

Jean Straus, Woman living with hearing loss

Masood Qureshi (MAQ), Lived experienced representative.

Peter Jones, Person living with hearing aids and dementia.

Prof. Tom Dening, Professor of Dementia Research, University of Nottingham, and Honorary Consultant in Old Age Psychiatry, Nottinghamshire Healthcare NHS Trust with personal experience of hearing loss.

Roulla Katiri, Clinical Research Audiologist, University College London and Advanced Audiologist, University College London Hospitals NHS Foundation Trust.

Ruth MacLeod, Health Policy Advisor, Royal National Institute for Deaf People (RNID)

Sarah Foster, Policy Manager, Alzheimer's Research UK (ARUK)

Sue Strachan, Lived experience representative, ARUK Ambassador
