# Supplementary material for: James Lind Alliance Priority Setting Partnership in co-existing dementia and hearing conditions: a research agenda defined by people with lived experience and healthcare professionals
Source: Age Ageing. 2025 Jul 6;54(7):afaf191. doi: 10.1093/ageing/afaf191 (PMC12229087; doi:10.1093/ageing/afaf191)
Supplement: Appendix_2_afaf191 [file appendix_2_afaf191.docx]

### **Appendix 2**

### **List of 47 Summary Research Questions**

Questions 1-16 appear in rank order, as decided at the final workshop. The remaining priority questions are presented in no particular order in accordance with the standard James Lind Alliance priority setting process.

1. What actions can people who have hearing loss take to reduce their risk of developing dementia?
2. Can the early detection and management of cognitive or hearing difficulties for people lead to better outcomes or treatments?
3. Does hearing loss increase dementia risk, and if so, what are the underlying mechanisms or causes (e.g. vascular disease, biological or neurological mechanisms)?
4. What training would help health professionals provide appropriate support and communicate effectively with people living with both dementia and hearing conditions?
5. Is the link between hearing loss and dementia risk impacted by other factors (e.g. personality, lifestyle, additional health conditions, or social isolation)?
6. Does dementia impact hearing, or does hearing impact dementia (e.g. severity, rate of progression)?
7. Can auditory and/or cognitive training be used by people with hearing loss to improve cognition and/or reduce the risk of developing dementia?
8. Should routine health checks in adults assess both hearing and cognition?
9. Are there heredity/genetical factors that increase the likelihood of developing co-existing dementia and hearing conditions?
10. What is the best way for primary care professionals (e.g. general practitioners) to support the assessment of dementia and hearing conditions and to improve their understanding of the link between them?
11. Can dementia risk be reduced by using hearing aids or cochlear implants, and if so, how important is it to start using them early?
12. Could collaboration between audiology services and other health and care services improve screening and diagnosis of dementia and/or hearing loss?
13. Does the duration, severity or type of hearing loss affect dementia risk?
14. Should people with hearing loss have their cognition assessed to help detect dementia earlier?
15. What is the likelihood of developing dementia in people living with hearing loss compared with the general population?
16. Is there a connection between dementia risk and hearing conditions other than hearing loss (e.g. tinnitus, hyperacusis)?

What are the benefits of an awareness campaign about the link between dementia and hearing loss for health and care professionals?

What are the benefits of an awareness campaign about the link between dementia and hearing loss for the general public?

For people living with dementia and/or hearing conditions, what are the most accurate screening and diagnostic tests for each condition?

What are the benefits of guidelines and training for audiologists about identifying, managing and referring patients who may have cognitive decline or dementia?

What is the best way for health professionals to discuss potential hearing loss with people living with dementia and their families?

How can health professionals differentiate between the symptoms of dementia and hearing conditions to support the diagnosis and management of each condition?

How many people with dementia, including those in care settings, have undiagnosed or untreated hearing loss?

Can screening for hearing loss in people living with dementia improve the management of both conditions?

How accessible are audiology services for people living with dementia?

How can people living with dementia and/or hearing conditions be best supported to accept their diagnoses and use recommended treatment or management options?

What is the impact of hearing conditions other than hearing loss (e.g. tinnitus, hyperacusis and balance disorders) on people living with dementia, and how best can this impact be managed?

At what age can the link between dementia and hearing loss be observed?

Is there an increased risk of developing dementia for people in the Deaf community, including Deaf people who use sign language?

How common is dementia amongst people who have had hearing loss since birth when compared with people who acquire hearing loss later in life?

What actions can members of the public take to reduce their risk of developing co-existing dementia and hearing conditions?

What is the economic impact of addressing hearing loss as a means of reducing dementia risk?

How can people living with dementia in care homes be best supported to manage their hearing loss?

What is the best way to train care home staff to help people living with co-existing hearing loss and dementia to manage hearing aids?

What support do carers need to help people living with co-existing dementia and hearing loss to manage hearing aids and other hearing treatments?

Are hearing aids the best hearing treatment for people living with co-existing hearing loss and dementia?

How can people with co-existing dementia and hearing loss be supported to manage their hearing loss with hearing aids?

Are there effective strategies, modifications, or adaptions to support hearing aid use for people living with dementia and hearing loss?

What hearing loss treatment or management options for people living with dementia are the most beneficial for their hearing and/or cognition?

What steps can people living with dementia take to prepare for or manage hearing loss, and can this help them maintain independence for longer?

Can hearing devices other than hearing aids be used to manage hearing loss in people living with dementia?

Do any of the medications given to people living with dementia lead to hearing conditions or vice versa?

For dementia risk reduction, how does the use of hearing aids and cochlear implants compare?

How can access to cochlear implants be improved for people living with co-existing dementia and hearing loss?

Are there any combined treatment or management options for both dementia and hearing conditions that improve outcomes in people living with both conditions?

What information and support do people living with co-existing dementia and hearing conditions and their carers need to manage both conditions?

How does the co-existence of dementia and hearing conditions impact the effectiveness of treatment or management options for each condition?
