# Supplementary material for: James Lind Alliance Priority Setting Partnership in co-existing dementia and hearing conditions: a research agenda defined by people with lived experience and healthcare professionals
Source: Age Ageing. 2025 Jul 6;54(7):afaf191. doi: 10.1093/ageing/afaf191 (PMC12229087; doi:10.1093/ageing/afaf191)
Supplement: Appendix_3_afaf191 [file appendix_3_afaf191.docx]

### **Appendix 3**

**Evidence Checking Process**

Below is a list of the 32 systematic reviews (1-32) and ten guidelines (33-42) identified and examined as part of the evidence-checking process. The 47 summary questions were checked against the evidence reported in these reviews and guidelines. All 47 questions were deemed unanswered. The primary reasons for these questions being deemed unanswered included: evidence did not provide definite conclusions with further research needed; the evidence centred on the topic of hearing and/or dementia but did not answer the specific summary questions; and the evidence did not focus on people living with both conditions.

1. Carvalho SC, Martins FS, Martins AN, et al.; Effectiveness of Snoezelen in older adults with neurocognitive and other pathologies: A systematic review of the literature. *Journal of neuropsychology* 2023(101468753). doi: <https://dx.doi.org/10.1111/jnp.12346>.

2. Buchholz S, Zempel H; The six brain-specific TAU isoforms and their role in Alzheimer's disease and related neurodegenerative dementia syndromes. *Alzheimer's & dementia : the journal of the Alzheimer's Association* 2024(101231978). doi: <https://dx.doi.org/10.1002/alz.13784>.

3. Borsetto D, Corazzi V, Obholzer R, et al.; Dizziness, psychological disorders and cognitive decline. *Panminerva medica* 2023;**65**(1):84-90. doi: <https://dx.doi.org/10.23736/S0031-0808.21.04209-9>.

4. Malesci R, Brigato F, Di Cesare T, et al.; Tinnitus and Neuropsychological Dysfunction in the Elderly: A Systematic Review on Possible Links. *Journal of clinical medicine* 2021;**10**(9). doi: <https://dx.doi.org/10.3390/jcm10091881>.

5. Hao J, Pu Y, He Z, et al.; Measurement properties of the backward walk test in people with balance and mobility deficits: A systematic review. *Gait & posture* 2024;**110**(9416830):1-9. doi: <https://dx.doi.org/10.1016/j.gaitpost.2024.02.018>.

6. Zhao Z, Yan J, Huang L, et al.; Phytochemicals targeting Alzheimer's disease via the AMP-activated protein kinase pathway, effects, and mechanisms of action. *Biomedicine & pharmacotherapy = Biomedecine & pharmacotherapie* 2024;**173**:116373-116373. doi: <https://dx.doi.org/10.1016/j.biopha.2024.116373>.

7. Mohanannair Geethadevi G, Quinn TJ, George J, et al.; Multi-domain prognostic models used in middle-aged adults without known cognitive impairment for predicting subsequent dementia. *The Cochrane database of systematic reviews* 2023;**6**(100909747):CD014885-CD014885. doi: <https://dx.doi.org/10.1002/14651858.CD014885.pub2>.

8. O'Neill M, Duffy O, Henderson M, et al.; Identification of eating, drinking and swallowing difficulties for people living with early-stage dementia: A systematic review. *International journal of language & communication disorders* 2023;**58**(6):1994-2007. doi: <https://dx.doi.org/10.1111/1460-6984.12924>.

9. Shao L, Shi Y, Xie X-Y, et al.; Incidence and Risk Factors of Falls Among Older People in Nursing Homes: Systematic Review and Meta-Analysis. *Journal of the American Medical Directors Association* 2023;**24**(11):1708-1717. doi: <https://dx.doi.org/10.1016/j.jamda.2023.06.002>.

10. Fu X, Eikelboom RH, Tian R, et al.; The Relationship of Age-Related Hearing Loss with Cognitive Decline and Dementia in a Sinitic Language-Speaking Adult Population: A Systematic Review and Meta-Analysis. *Innovation in aging* 2023;**7**(1):igac078-igac078. doi: <https://dx.doi.org/10.1093/geroni/igac078>.

11. Samara M, Thai-Van H, Ptok M, et al.; A systematic review and metanalysis of questionnaires used for auditory processing screening and evaluation. *Frontiers in neurology* 2023;**14**(101546899):1243170-1243170. doi: <https://dx.doi.org/10.3389/fneur.2023.1243170>.

12. Ilut S, Vesa SC, Vacaras V, et al.; Biological Risk Factors Influencing Vascular Cognitive Impairments: A Review of the Evidence. *Brain sciences* 2023;**13**(7). doi: <https://dx.doi.org/10.3390/brainsci13071094>.

13. Bahji A, Breward N, Duff W, et al.; Cannabinoids in the management of behavioral, psychological, and motor symptoms of neurocognitive disorders: a mixed studies systematic review. *Journal of cannabis research* 2022;**4**(1):11-11. doi: <https://dx.doi.org/10.1186/s42238-022-00119-y>.

14. Kiper P, Richard M, Stefanutti F, et al.; Combined Motor and Cognitive Rehabilitation: The Impact on Motor Performance in Patients with Mild Cognitive Impairment. Systematic Review and Meta-Analysis. *Journal of personalized medicine* 2022;**12**(2). doi: <https://dx.doi.org/10.3390/jpm12020276>.

15. Marin-Jimenez N, Cruz-Leon C, Perez-Bey A, et al.; Predictive Validity of Motor Fitness and Flexibility Tests in Adults and Older Adults: A Systematic Review. *Journal of clinical medicine* 2022;**11**(2). doi: <https://dx.doi.org/10.3390/jcm11020328>.

16. Gommeren H, Bosmans J, Cardon E, et al.; Cortical Auditory Evoked Potentials in Cognitive Impairment and Their Relevance to Hearing Loss: A Systematic Review Highlighting the Evidence Gap. *Frontiers in neuroscience* 2021;**15**(101478481):781322-781322. doi: <https://dx.doi.org/10.3389/fnins.2021.781322>.

17. Bisogno A, Scarpa A, Di Girolamo S, et al.; Hearing Loss and Cognitive Impairment: Epidemiology, Common Pathophysiological Findings, and Treatment Considerations. *Life (Basel, Switzerland)* 2021;**11**(10). doi: <https://dx.doi.org/10.3390/life11101102>.

18. Ryan D, Fullen B, Rio E, et al.; Effect of Action Observation Therapy in the Rehabilitation of Neurologic and Musculoskeletal Conditions: A Systematic Review. *Archives of rehabilitation research and clinical translation* 2021;**3**(1):100106-100106. doi: <https://dx.doi.org/10.1016/j.arrct.2021.100106>.

19. Yan J, Li X, Guo X, et al.; Effect of Multicomponent Exercise on Cognition, Physical Function and Activities of Daily Life in Older Adults With Dementia or Mild Cognitive Impairment: A Systematic Review and Meta-analysis. *Archives of physical medicine and rehabilitation* 2023;**104**(12):2092-2108. doi: <https://dx.doi.org/10.1016/j.apmr.2023.04.011>.

20. Tuena C, Borghesi F, Bruni F, et al.; Technology-Assisted Cognitive Motor Dual-Task Rehabilitation in Chronic Age-Related Conditions: Systematic Review. *Journal of medical Internet research* 2023;**25**(100959882):e44484-e44484. doi: <https://dx.doi.org/10.2196/44484>.

21. O'Brien J, Mason A, Chan J, et al.; Can We Train Multisensory Integration in Adults? A Systematic Review. *Multisensory research* 2023;**36**(2):111-180. doi: <https://dx.doi.org/10.1163/22134808-bja10090>.

22. Wen Z, Peng S, Yang L, et al.; Factors Associated With Social Isolation in Older Adults: A Systematic Review and Meta-Analysis. *Journal of the American Medical Directors Association* 2023;**24**(3):322-330.e6. doi: <https://dx.doi.org/10.1016/j.jamda.2022.11.008>.

23. Hooper E, Brown LJE, Cross H, et al.; Systematic Review of Factors Associated With Hearing Aid Use in People Living in the Community With Dementia and Age-Related Hearing Loss. *Journal of the American Medical Directors Association* 2022;**23**(10):1669-1675.e16. doi: <https://dx.doi.org/10.1016/j.jamda.2022.07.011>.

24. Yang T, Zhang F; Targeting Transcription Factor Nrf2 (Nuclear Factor Erythroid 2-Related Factor 2) for the Intervention of Vascular Cognitive Impairment and Dementia. *Arteriosclerosis, thrombosis, and vascular biology* 2021;**41**(1):97-116. doi: <https://dx.doi.org/10.1161/ATVBAHA.120.314804>.

25. Tsai YIP, Browne G, Inder KJ; The effectiveness of interventions to improve pain assessment and management in people living with dementia: A systematic review and meta-analyses. *Journal of advanced nursing* 2021;**77**(3):1127-1140. doi: <https://dx.doi.org/10.1111/jan.14660>.

26. Chantanachai T, Sturnieks DL, Lord SR, et al.; Risk factors for falls in older people with cognitive impairment living in the community: Systematic review and meta-analysis. *Ageing research reviews* 2021;**71**(101128963):101452-101452. doi: <https://dx.doi.org/10.1016/j.arr.2021.101452>.

27. Davies N, Barrado-Martin Y, Vickerstaff V, et al.; Enteral tube feeding for people with severe dementia. *The Cochrane database of systematic reviews* 2021;**8**(100909747):CD013503-CD013503. doi: <https://dx.doi.org/10.1002/14651858.CD013503.pub2>.

28. Cross H, Dawes P, Hooper E, et al.; Effectiveness of Hearing Rehabilitation for Care Home Residents With Dementia: A Systematic Review. *Journal of the American Medical Directors Association* 2022;**23**(3):450-460.e4. doi: <https://dx.doi.org/10.1016/j.jamda.2021.11.011>.

29. Meng HYH, Mak CCH, Mak WY, et al.; Probiotic supplementation demonstrates therapeutic potential in treating gut dysbiosis and improving neurocognitive function in age-related dementia. *European journal of nutrition* 2022;**61**(4):1701-1734. doi: <https://dx.doi.org/10.1007/s00394-021-02760-4>.

30. Tarawneh HY, Menegola HK, Peou A, et al.; Central Auditory Functions of Alzheimer's Disease and Its Preclinical Stages: A Systematic Review and Meta-Analysis. *Cells* 2022;**11**(6). doi: <https://dx.doi.org/10.3390/cells11061007>.

31. Li F, Wang L, Qin Y, et al.; Combined Tai Chi and cognitive interventions for older adults with or without cognitive impairment: A meta-analysis and systematic review. *Complementary therapies in medicine* 2022;**67**(9308777):102833-102833. doi: <https://dx.doi.org/10.1016/j.ctim.2022.102833>.

32. Chari DA, Madhani A, Sharon JD, et al.; Evidence for cognitive impairment in patients with vestibular disorders. *Journal of neurology* 2022;**269**(11):5831-5842. doi: <https://dx.doi.org/10.1007/s00415-022-11289-3>.

33. National Institute for Health and Care Excellence; Dementia: assessment, management and support for people living with dementia and their carers 2018.

34. National Institute for Health and Care Excellence; Dementia, disability and frailty in later life – mid-life approaches to delay or prevent onset 2015.

35. National Institute for Health and Care Excellence; Dementia. 2019.

36. National Institute for Health and Care Excellence; Hearing loss in adults: assessment and management 2023.

37. National Institute for Health and Care Excellence; Tinnitus: assessment and management. 2020.

38. National Institute for Health and Care Excellence; Cochlear implants for children and adults with severe to profound deafness 2019.

39. National Institute for Health and Care Excellence; Multimorbidity: clinical assessment and management 2016.

40. National Institute for Health and Care Excellence; Older people with social care needs and multiple long-term conditions. 2015.

41. Scottish Intercollegiate Guidelines Network; Assessment, diagnosis, care and support for people with dementia and their carers 2023.

42. Royal College of Psychiatrists; National Audit on Dementia. 2022.
